# AI-Driven Antimicrobial Peptide Discovery: Mining and Generation

**DOI:** 10.1021/acs.accounts.0c00594

**Published:** 2025-06-03

**Authors:** Paulina Szymczak, Wojciech Zarzecki, Jiejing Wang, Yiqian Duan, Jun Wang, Luis Pedro Coelho, Cesar de la Fuente-Nunez, Ewa Szczurek

**Affiliations:** † Institute of AI for Health, 685889 Helmholtz Zentrum Munich, Neuherberg 85764, Germany; ‡ Faculty of Mathematics, Informatics and Mechanics, 121883 University of Warsaw, Warsaw 02-097, Poland; § Faculty of Electronics and Information Technology, Warsaw University of Technology, Warsaw 00-661, Poland; ∥ Institute of Microbiology, 12381 Chinese Academy of Sciences; Beijing 100101, China; ⊥ Institute of Science and Technology for Brain-Inspired Intelligence, 600441 Fudan University, Shanghai 200433, China; # Centre for Microbiome Research, School of Biomedical Sciences, Queensland University of Technology, Translational Research Institute, Woolloongabba, Queensland 4102, Australia; 7 Centre for Data Science, Queensland University of Technology, Brisbane, Queensland 4001, Australia; 8 Machine Biology Group, Departments of Psychiatry and Microbiology, Institute for Biomedical Informatics, Institute for Translational Medicine and Therapeutics, Perelman School of Medicine, University of Pennsylvania, Philadelphia, Pennsylvania 19104, United States of America; 9 Departments of Bioengineering and Chemical and Biomolecular Engineering, School of Engineering and Applied Science, University of Pennsylvania, Philadelphia, Pennsylvania 19104, United States of America; 10 Department of Chemistry, School of Arts and Sciences, 6572 University of Pennsylvania, Philadelphia, Pennsylvania 19104, United States of America; 11 Penn Institute for Computational Science, University of Pennsylvania, Philadelphia, Pennsylvania 19104, United States of America

## Abstract

The escalating threat of antimicrobial resistance (AMR) poses a significant global health crisis, potentially surpassing cancer as a leading cause of death by 2050. Traditional antibiotic discovery methods have not kept pace with the rapidly evolving resistance mechanisms of pathogens, highlighting the urgent need for novel therapeutic strategies. In this context, antimicrobial peptides (AMPs) represent a promising class of therapeutics due to their selectivity toward bacteria and slower induction of resistance compared to classical, small molecule antibiotics. However, designing effective AMPs remains challenging because of the vast combinatorial sequence space and the need to balance efficacy with low toxicity. Addressing this issue is of paramount importance for chemists and researchers dedicated to developing next-generation antimicrobial agents.

Artificial intelligence (AI) presents a powerful tool to revolutionize AMP discovery. By leveraging AI, we can navigate the immense sequence space more efficiently, identifying peptides with optimal therapeutic properties. This Account explores the emerging application of AI in AMP discovery, focusing on two primary strategies: AMP mining, and AMP generation, as well as the use of discriminative methods as a valuable toolbox.

AMP mining involves scanning biological sequences to identify potential AMPs. Discriminative models are then used to predict the activity and toxicity of these peptides. This approach has successfully identified numerous promising candidates, which were subsequently validated experimentally, demonstrating the potential of AI in AMP design and discovery.

AMP generation, on the other hand, creates novel peptide sequences by learning from existing data through generative modeling. This class of models optimizes for desired properties, such as increased activity and reduced toxicity, potentially producing synthetic peptides that surpass naturally occurring ones. Despite the risk of generating unrealistic sequences, generative models hold the promise of accelerating the discovery of highly effective and highly novel and diverse AMPs.

In this Account, we describe the technical challenges and advancements in these AI-based approaches. We discuss the importance of integrating various data sources and the role of advanced algorithms in refining peptide predictions. Additionally, we highlight the future potential of AI to not only expedite the discovery process but also to uncover peptides with unprecedented properties, paving the way for next-generation antimicrobial therapies.

In conclusion, the synergy between AI and AMP discovery opens new frontiers in the fight against AMR. By harnessing the power of AI, we can design novel peptides that are both highly effective and safe, offering hope for a future where AMR is no longer a looming threat. Our paper underscores the transformative potential of AI in drug discovery, advocating for its continued integration into biomedical research.

## Graphical abstract

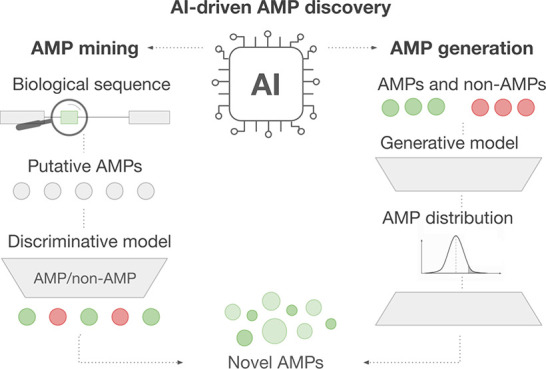

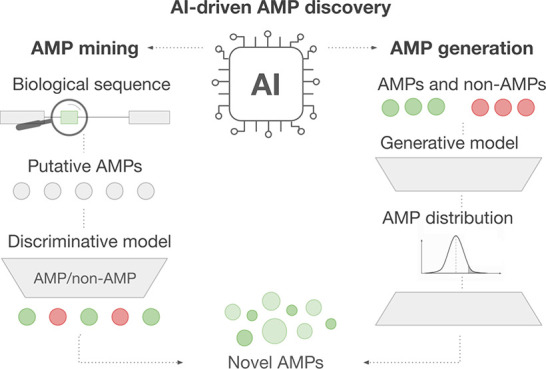

## Key references

-

Szymczak, P. ; Możejko, M. ; Grzegorzek, T. et al. Discovering highly potent antimicrobial peptides with deep generative model HydrAMP. Nat. Commun. 2023, 14, 1453 36922490 10.1038/s41467-023-36994-zPMC10017685.^1^ *Extended conditional variational autoencoder, trained for unconstrained and analogue generation of novel active AMPs, with parametrized creativity*.
-

Wan, F. ; Torres, M. D. T. ; Peng, J. ; de la Fuente-Nunez, C. Deep-learning-enabled antibiotic discovery through molecular de-extinction. Nat. Biomed. Eng. 2024, 8, 854–871 38862735 10.1038/s41551-024-01201-xPMC11310081.^2^ *This paper introduced APEX, a deep learning model used to mine all extinct organisms known to science as a source of antibiotic molecules, leading to the identification of preclinical candidates such as mammuthusin and elephasin.*
-

Santos-Júnior, C. D. ; Torres, M. D. T. ; Duan, Y. ; Rodríguez Del Río, Á. ; Schmidt, T. S. B. ; Chong, H. ; Fullam, A. ; Kuhn, M. ; Zhu, C. ; Houseman, A. ; Somborski, J. ; Vines, A. ; Zhao, X. M. ; Bork, P. ; Huerta-Cepas, J. ; de la Fuente-Nunez, C. ; Coelho, L. P. Discovery of antimicrobial peptides in the global microbiome with machine learning. Cell 2024, 187, P3761-3778.E16 10.1016/j.cell.2024.05.013PMC1166632838843834.^3^ *Nearly one million candidate peptide antibiotics identified in the global microbiome with discriminative machine learning*.
-

Torres, M. D. T. ; Melo, M. C. R. ; Flowers, L. ; Crescenzi, O. ; Notomista, E. ; de la Fuente-Nunez, C. Mining for encrypted peptide antibiotics in the human proteome. Nat. Biomed. Eng. 2022, 6, 67–75 34737399 10.1038/s41551-021-00801-1.^4^ *First exploration of the human proteome as a source of antibiotics, revealing thousands of previously unrecognized peptides, many of which may contribute to host immunity and other physiological processes.*
-

Ma, Y. ; Guo, Z. ; Xia, B. ; Zhang, Y. ; Liu, X. ; Yu, Y. ; Tang, N. ; Tong, X. ; Wang, M. ; Ye, X. ; Feng, J. ; Chen, Y. ; Wang, J. Identification of antimicrobial peptides from the human gut microbiome using deep learning. Nat. Biotechnol. 2022, 40, 921–931 35241840 10.1038/s41587-022-01226-0.^5^ *Expanding the deep learning methods for AMP discovery to anticancer peptides*.

## Introduction

Following the boom of antibiotic discoveries in the 20th century, the past three decades have experienced a discovery void, with no novel antibiotic classes reaching the market.^6^ Concurrently, resistance to existing antibiotics has escalated.^7^ Antimicrobial resistance has become a significant global health and economic issue, projected to surpass cancer as a leading cause of death by 2050.^8^ The antibiotic discovery process remains cumbersome, often requiring many years to identify preclinical candidates, resulting in the lack of antibiotic innovation.^9–11^

A promising strategy to combat antimicrobial resistance involves designing novel antimicrobial peptides (AMPs). These peptides are typically short (10–100 amino acids), with a net positive charge (commonly +2 to +9) and a high proportion (≥30%) of hydrophobic amino acids. Positively charged AMPs show distinct selectivity for negatively charged microbial membranes and tend not to target the neutral membranes of eukaryotic cells. Other than membrane targeting, modes of AMP action include inhibition of key processes such as protein or nucleic acid synthesis, protease activity or cell division. Microbial targets and modes of action of AMPs are determined by their amino acid composition and structure, with distinguished AMP classes including proline-rich, tryptophan- and arginine, histidine or glycine-rich peptides, majority of which are alpha helical but may also adopt beta-sheet, linear extension or mixed alpha-beta conformations. AMPs play important roles in host response to pathogen infection in a wide range of organisms, and are found in mammals, amphibia, insects, and microorganisms.^12^ Importantly, microbes develop resistance to AMPs more slowly than to traditional antibiotics.^13^ However, many AMPs exhibit toxicity to mammalian cells, often assessed through cytotoxic and hemolytic activities.^14^ While multiple characteristics such as solubility and stability are important for AMPs,^15^ the primary challenge remains in designing peptides that are highly active and minimally toxic. Given the limited success of known peptides in the clinic, innovative methods for designing AMP are essential to enhance their properties beyond those of existing peptides.^15,16^

The design of AMPs could be defined as an optimization problem, namely as searching for the most active and least toxic peptides within a vast space of potential sequences, with only scarce data on their properties. Considering sequences of up to 25 amino acids in length, a brute-force search algorithm would need to evaluate on the order of 10^32^ sequences, a task that is computationally infeasible.^17^ In contrast, the number of experimentally verified AMPs (on the order of 10^4^, based on the DBAASP database^18^) is minuscule. Peptides with documented activity against specific bacterial species like *Escherichia coli* (*E. coli)* are even scarcer (around 10^3^).

To navigate this vast search space effectively, sophisticated algorithms are necessary. These algorithms must strike a balance between selecting *realistic* peptide sequences, i.e. those that resemble existing AMPs, and designing *idealistic* peptides, i.e., optimizing peptides for heightened activity and low toxicity, resulting in a *realism-idealism trade-off*. Advances in AI-driven AMP design revealed two primary strategies: (i) biological sequence mining, and (ii) generative AI. AMP mining identifies peptides by exploring genomes and proteomes and evaluating potential candidates using discriminative models that predict their activity or toxicity. This approach has successfully yielded peptides that are likely to be naturally produced, aligning with the realism aspect of the design. Conversely, generative AI models learn the distribution of peptide data and generate novel sequences, often optimizing them with predictive models to enhance activity and reduce toxicity. This strategy is capable of creating idealistic, synthetic peptides, potentially exceeding those found in nature, though it risks producing sequences that may not be sufficiently realistic. Both genome mining and generative AI approaches have the potential to dramatically accelerate antibiotic discovery, enabling the identification of hundreds of thousands of potential candidate molecules. Indeed, the AI-based algorithms developed so far have successfully designed and discovered peptides, some with proven efficacy in preclinical mouse models.^2–5,19,20^

Here, we describe recent advancements and the current state of the art in discriminative methods for assessing activity and toxicity, which are often instrumental for AI-driven AMP design. This is followed by a systematic review on the emerging areas of AMP mining and generative AI-based strategies for AMP discovery. Finally, we discuss remaining challenges in AMP design and outline promising research directions.

## Discriminative Methods

Discriminative methods serve for both AMP mining and AMP generation, and are crucial for the selection of promising active and nontoxic candidates. The majority of models broadly distinguish AMPs from non-AMPs (e.g., sAMP-pred-GAT,^21^ AMPlify,^22^ and AMPpredMFA^23^). More elaborate approaches focus on identifying highly potent peptides either via classification or regression by incorporating information on MIC measurements into the model.^24,25^ Strain- or species-specific discriminators attempt to select peptides with an activity profile specific to a given microbe, such as AMP-META,^26^ or MBC-attention.^24^ While much less popular due to data scarcity, approaches for AMP toxicity exist as well, such as EnDL-HemoLyt,^27^ AMP-META,^26^ Macrel^28^ and others.^29–32^ Strikingly, only few of the discriminative models are evaluated experimentally through microbiological assays, and even less frequently through hemolytic activity and cytotoxicity assays. An overview of recent discriminative methods summarizing employed frameworks, feature types, performed tasks, and experimental validation is provided in Table 1.

**Table 1:** Discriminative Methods for AMP Discovery^a^

method	framework	feature type	task	experimental validation	approach type
sAMPpred-GAT^21^	GNN, ATT; MLP	sequence-derived descriptors, structure	AMP		ML-based
AMPlify^22^	LSTM, ATT; MLP	sequence	AMP	microbiological assays	
AMPpredMFA^23^	LSTM, CNN, ATT; MLP	sequence	AMP		
MBC-attention^24^	CNN, ATT; MLP	sequence derived	activity		
AMP-META^26^	LGBM	structure, sequence-derived descriptors	AMP, activity, toxicity	microbiological assays	
EnDL-HemoLyt^27^	LSMT, CNN; MLP	sequence	toxicity		
Macrel^28^	RF	sequence-derived descriptors	AMP, toxicity		
Pandi et al^24^	CNN, RNN; MLP	sequence	activity	microbiological assays, hemolysis assays, cytotoxicity assays	
APEX^2^	RNN, ATT; MLP	sequence	activity	microbiological assays, in vivo animal models, cytotoxicity assays	
Capecchi et al.^29^	RNN, GRU, SVM; MLP	sequence	activity, toxicity	microbiological assays, hemolysis assays	
Ansari and White^32^	RNN, LSTM	sequence	toxicity, solubility		
ESKAPEE-MICpred^31^	LSTM, CNN; MLP	sequence, sequence-derived descriptors	activity	microbiological assays	
Ansari and White^30^	LSTM; MLP	sequence	toxicity, non-fouling activity, SHP-2		
Zhuang and Shengxin^38^	QSVM	sequence-derived descriptors	toxicity		
AmPEPpy^34^	RF	sequence	AMP		
Orsi and Reymond^46^	GPT-3; MLP	sequence	toxicity, solubility		LLM-based
iAMP-Attenpred^40^	BERT; MLP	pLM embedding	AMP		
PepHarmony^41^	ESM, GearNet; MLP	sequence, structure	solubility, affinity, self-contaction		
SenseXAMP^42^	ESM-1b; MLP	pLM embedding	activity		
HDM-AMP^43^	ESM-1b; DF	pLM embedding	activity	microbiological assays	
AMPFinder^51^	ProtTrans, OntoProtein; MLP	pLM embedding	activity		
LMPred^52^	ProtTrans; MLP	pLM embedding	activity		
PHAT^49^	ProtTrans; MLP	pLM embedding	secondary structure		
PeptideBERT^47^	BERT (ProtBert); MLP	pLM embedding	toxicity, solubility, non-fouling activity		
TransImbAMP^53^	BERT; MLP	pLM embedding	activity		
AMPDeep^45^	BERT (ProtBert); MLP	pLM embedding	toxicity		
Zhang et al.^48^	BERT; MLP	pLM embedding	activity		
Ma, Yue, et al^5^	BERT, ATT, LSTM; MLP	sequence	AMP	microbiological assays, in vivo animal models, hemolysis assays, cytotoxicity assays	
iAMP-CA2L^39^	CNN, Bi-LSTM, MLP; SVM	structure	AMP		structure-based
sAMP-VGG16 ^55^	CNN; MLP	sequence-derived descriptors	AMP		
AMPredictor^56^	ESM; MLP	sequence-derived descriptors, structure	activity	microbiological assays, in vivo animal models, hemolysis assays	

a GNN: Graph Neural Network; ATT: attention mechanism, MLP: Multi-layer perceptron, LSTM: Long Short-Term Memory, CNN: Convolutional Neural Network, LGBM: Light Gradient-Boosting Machine, RF: Random Forest, RNN: Recurrent Neural Network, GRU: Gated Recurrent Unit, SVM: Supporting Vector Machine, QSVM: Quantum Supporting Vector Machine, GPT-3: Generative Pre-trained Transformer 3, BERT: Bidirectional Encoder Representations from Transformers, ESM: Evolutionary Scale Modeling, DF: Deep Forest, Bi-LSTM: Bi-directional Long Short-Term Memory.

### Models and Architectures Applied for the Development of Discriminative Methods

Traditional ML methods, such as decision trees, Support Vector Machines (SVMs), and random forest (RF), rely entirely on sequence-derived descriptors for AMP prediction.^33,34^ Importantly, because of their simplicity, these methods can be employed to infer biological insights, such as analysis of Shapley Additive exPlanations to reveal differences in AMP mechanisms of action between Gram-negative and Gram-positive bacteria.^26^ Another example of a traditional ML discriminator for AMPs is Macrel,^28^ a random forest model, trained on an unbalanced data set containing a ratio of approximately 1:50 of AMPs vs non-AMPs to more closely mimic the expected distribution in a genome mining task than a balanced data set. Macrel was successfully applied in a recent large AMP mining study, AMPSphere.^3^ The relative simplicity and on-par or, for some tasks, better performance than more sophisticated, deep-learning (DL) based approaches make traditional ML methods a recommendable choice for AMP identification.^35^

Still, compared to traditional machine learning methods, DL models have the potential to increase effectiveness in addressing more complex challenges and improve prediction accuracy for AMP discrimination.^36,37^ The most ubiquitous DL methods in AMP prediction are derived from models originally devised for natural languages, such as recurrent neural networks (RNNs),^19,25,29^ or long short-term memory (LSTM) architectures.^25,30–32^ Furthermore, attention mechanisms have emerged as a pivotal component of many recent architectures. Through examination of features related to sequence composition, such as frequency of occurrence of each amino acid, and backward and forward relationships, these models gain a profound comprehension of the ″semantics″ inherent in biological sequences. Models such as AMPlify^22^ achieve that by incorporating autoregressive models like Bi-LSTM and attention layers. Although convolutional neural networks (CNNs) were originally introduced for vision-related tasks, they are also used for AMP prediction, based on sequence-derived features. One example of a CNN-based model is MBC-Attention,^24^ which combines multibranch CNN with attention mechanism to regress the minimum inhibitory concentration of AMPs against *E. coli.* An approach combining the strengths of both autoregressive and CNN-based methods is AMPpred-MFA,^23^ which uses both bidirectional-LSTMs and CNNs, followed by multihead attention mechanism to extract context dependencies of peptide sequences. Finally, a quantum supporting vector machine was proposed by Zhuang and Shengxin to detect toxicity of peptides based on sequence derived descriptors.^38^

### Large Language Models Applied in Discriminative Methods

While deep learning networks such as RNNs, LSTMs or CNNs do take into account the context relationships within amino acids, the recent large language models (LLMs) based on the transformer architecture offer novel opportunities for analysis of large corpuses of sequence data, in particular by efficiently leveraging the attention mechanism. In particular, LLMs have been successfully applied to protein sequences, resulting in so-called protein language models (PLMs).^39^ The process of training such models usually follows two steps. First, a transformer model is pretrained on a large corpus of proteins in a generative task, and then fine-tuned to a specific downstream task, such as function, property, or structure prediction. Similarly to ML-based approached, PLMs have been applied to predict antimicrobial activity^5,40–45^ and nontoxicity,^46–48^ but other properties such as solubility or secondary structure^41,46,47,49^ have been tackled as well (Table 1).

Compared to typical proteins, peptides are shorter in length and have relatively less complex tertiary structure. Moreover, the number of known bioactive peptides is much smaller than the number of known proteins, and the number of AMPs validated by experimental methods is limited. Therefore, direct application of PLMs without additional fine-tuning to AMP data would result in models biased toward more protein-like properties. Indeed, models trained on proteins, ‘chopped proteins’ (short subsequences of proteins) and peptides result in different embeddings of the input sequences and the models trained on shorter sequences have more generalized embeddings and perform better in downstream tasks.^50^ While attempts to directly use text-based pretrained LLMs without additional pretraining on protein corpus have been made,^40,46^ this approach was shown to result in inferior performance to RNNs^46^ as the embeddings trained on natural text are likely not suitable for the domain of peptide sequences.

The most prevalently used LLM architectures are bidirectional encoder representation from transformers (BERT), which are effective in dealing with long-distance dependencies and thus learn the global context information on input sequences. Apart from such BERT-based models, other encoder-only architectures are successfully employed for AMP classification, in particular Evolutionary Scale Modeling (ESM) encoders, built upon the concept of integrating both sequence and evolutionary information.^41–43^ OntoProtein, a BERT-like model based on both protein sequences and the gene ontology (GO), was incorporated into AMPFinder^51^ to predict the functional types of AMP. However, in a recent evaluation by Dee,^52^ full encoder-decoder transformer architectures^49,51,52^ were proven to outperform the encoder-only models, confirming the results of Elnaggar et al., who performed similar benchmarking for proteins.^44^

Apart from architecture, the PLM models in AMP prediction differ also by their pretraining corpus, with most methods using UniRef50,^41–43,49,51,52^ fewer using UniRef100,^47,51^ and individual cases pretraining on Pfam,^53^ BFD,^45,52^ and UniProt,^48^ or merging corpuses.^51^ The selection of the pretraining corpus has a significant influence on model performance, as more diverse corpuses, such as UniRef50 having lower between-sequence similarities than UniRef100, were shown to improve results without any changes to the architecture.^52^

While most models directly proceed with training by adding prediction heads to the pretrained models and fine-tuning for discriminative AMP tasks, some approaches incorporate an additional phase of fine-tuning beforehand, for example using secretory data as an additional corpus^45^ for toxicity prediction or data for sequences shorter than 50 amino acids.^41^ Such additional pretraining phases may shift the pretrained model’s focus toward distributions of sequences that are more peptide- or AMP-like. Indeed, as peptides are much shorter and with simpler structure than proteins, LLMs pretrained on proteins may not adequately represent the peptide distribution.^50^

### Representations of Peptides Used by Discriminative Methods

Various discriminative models differ by the representation of peptides as their input features. The most prevalent representation is the amino acid sequence. While it can serve as a primary input to the model, it is also used to obtain sequence-derived descriptors or embeddings from pretrained models. Feature encoding using PLMs outperformed human-engineered features in a benchmarking study of García-Jacas et al.^54^ Still, Zhang et al. improved the performance of their model SenseXAMP^42^ in AMP prediction by fusing the embedding of a pretrained protein model with traditional protein descriptors (PD). SenseXAMP performed better than simply fine-tuning pretrained models, indicating that traditional PDs continue to play a crucial role in AMP screening tasks. Other approaches convert a sequence to an image, either using cellular automata^40^ or atom connectivity information,^55^ and then apply CNNs as the architecture for discriminative models.

Apart from methods that focus on amino acid sequence as the primary peptide representation, some methods try to incorporate structural information as an additional, complementary view. Of particular interest are methods that leverage graph-based approaches to encode structural information about peptides. For example, sAMP-pred-GAT^21^ integrates structural, sequence, and evolutionary information on peptides to construct a graph attention network (GAT) used to identify AMPs. Similarly, AMPredictor^56^ is a Graph Convolutional Net that incorporates Morgan fingerprints, peptide contact maps, and embedding from ESM to regress MIC values. Graph encoding was also combined with pLM embedding in PepHarmony,^41^ which merges sequence-level encoding from ESM with structure-level embedding from GearNet in multiview contrastive learning.

## AMP Mining

The availability of biological sequence data has seen an unprecedented expansion in recent years sparking efforts to discover new AMPs using mining strategies. AMP mining involves applying the previously discussed discriminative methods to biological sequence data, including genomes, proteomes, and metagenomes. Historically, AMPs were oftentimes found in the skin secretions from amphibians.^57^ While AMP mining requires careful handling to minimize false positives, particularly given the very large size of input data sets, it can yield high-quality predictions that have been substantiated through both *in vitro* and *in vivo* validations.^3,5,58^ The AMP mining approaches primarily target antimicrobial properties and they differ by the type and source of analyzed biological sequence collections (Table 2). Majority of AMP mining methods do not rely on toxicity predictors, likely due to their low reliability.

**Table 2:** Mining approaches for AMP discovery

approach	tool applied	model	biological sequence type	biological sequence source	experimental validation	mining criteria
Torres et al.^4^	Pane *et* ^77^	fitness function	proteome	*Homo sapiens*	microbiological assays, *in vivo* animal models	activity
Cesaro et al.^66^	Pane et al.^77^	fitness function	proteome	*Homo sapiens*	microbiological assays, *in vivo* animal models, cytotoxicity assays	activity
APEX^2^	APEX^2^	RNN, ATT; MLP	proteome	Extinct species	microbiological assays, *in vivo* animal models, cytotoxicity assays	activity
Torres et al.^68^	Pane et al.^77^	fitness function	proteome	*Homo sapiens*	microbiological assays, *in vivo* animal models, cytotoxicity assays	activity
panCleave^19^	panCleave^19^	random forest	proteome	extinct species	microbiological assays, *in vivo* animal models	AMP
Monsalve et al.^67^	PepMultiFinder, CAMPR3, AMP Scanner and AmpClass 1.0		proteome	eight species of bacteria, plants, a protist, and a nematode	microbiological assays, cytotoxicity assays, hemolysis assays	AMP
Klimovich and Bosch^73^	HMMER, BLAST		genome, proteome, transcriptome	*Hydra* microbiome		AMP orthologs
Ma et al.^5^	Ma et al.^5^	Ensemble of LSTM, attention and BERT	genome, proteome	*Homo sapiens* microbiome	microbiological assays, *in vivo* animal models, cytotoxicity assays, hemolysis assays	AMP
Ma et al.^72^	Ma et al.^5^	Ensemble of LSTM, attention and BERT	genome	*Homo sapiens* microbiome	microbiological assays, *in vivo* animal models, cytotoxicity assays, anticancer activity assays	AMP, ACP
Torres et al.^34^	SmORFinder, AmPEPpy		genome	*Homo sapiens* microbiome	microbiological assays, *in vivo* animal models, cytotoxicity assays	AMP/activity
Santos-Júnior et al.^28^	Macrel^28^	random forest	genome, proteome, transcriptome	global microbiome		AMP/activity, nontoxicity
GMSC-mapper^60^	GMSC-mapper^60^		genome	global microbiome		smORFs
Wu et al.^71^	Ma et al.^5^	ensemble of LSTM, attention and BERT	genome	ESKAPE + ESKAPE phages		AMP/activity
SMEP^75^	SMEP^75^	XGBoost, LSTM	hexapeptide, heptapeptide, octapeptide and nonapeptide libraries		microbiological assays, *in vivo* animal models, cytotoxicity assays, hemolysis assays	activity
FSLSMEP^78^	FSLSMEP^78^	ESM-1, MLP	hexapeptides, heptapeptides and octapeptides libraries		microbiological assays, *in vivo* animal models, cytotoxicity assays, hemolysis assays	activity

### Biological Sequence Collections Amenable for AMP Mining

Today, millions of genomes are accessible, and, together with metagenomes (which contain the genomic material of multiple organisms in a microbial community) or proteomes, they are collected in public databases.^59–63^ An example of rich data is the nonredundant Global Microbial Gene Catalogue (GMGCv1), created from thousands of metagenomes across numerous habitats. This resource includes billions of open reading frames (ORFs) clustered at a high nucleotide identity level, resulting in hundreds of millions of species-level unigenes.^64^ The GMGCv1 also contains tens of thousands of AMR genes, identified through homology-based searches against the Comprehensive Antibiotic Resistance Database (CARD)^65^ and alignment with known resistance gene sequences. In another study, Duan and colleagues constructed a global microbial catalog of small open reading frames (smORFs), which encode small proteins. The catalog, named GMSC, was derived from thousands of publicly available metagenomes across multiple distinct habitats and thousands of high-quality isolate genomes. GMSC contains close to a million nonredundant smORFs with comprehensive annotations and provides a tool called GMSC-mapper to identify and annotate small proteins from microbial (meta)­genomes.^60^

### AMP Mining of Genomes and Proteomes

Recently, the human proteome was explored as a source of antibiotics.^4,66–68^ The landmark study of Torres et al. employed an algorithm that utilized key physicochemical properties such as sequence length, net charge, and average hydrophobicity to predict antimicrobial activity. Building on the work of Pane et al.,^77^ this algorithm models antimicrobial potency as being linearly dependent on physicochemical properties raised to exponents - model parameters that were fitted using known AMPs. Specifically, the algorithm scanned 42,361 protein sequences from the human proteome and identified 2,603 potential AMP candidates, many of which were previously unrecognized as antimicrobials or to play a role in host immunity. By avoiding known AMP motifs and focusing on physicochemical characteristics, these explorations led to the discovery of novel antimicrobials, several of which were synthesized, validated experimentally, and showed efficacy in animal models.

AI has also enabled biological mining efforts^69^ to explore proteins from extinct species, such as Neanderthals and Denisovans, revealing a new set of antimicrobial sequences and launching the field of molecular de-extinction.^19^ In this work, the authors introduced panCleave - a random forest model for proteome-wide cleavage site prediction. For selection of candidate AMPs, apart from expert curation, they used a consensus of six publicly available traditional ML-based AMP models, including Macrel.^28^ Another study mined the proteomes of all available extinct organisms, including the woolly mammoth. Using a more powerful deep learning model called APEX,^2^ this study led to the discovery of novel AMPs, such as neanderthalin-1, mammuthusin-2, and elephasin-2, which now represent preclinical candidates. These computational efforts have drastically accelerated our ability to discover new antibiotics, transforming a process that once took years into one that can be completed in hours.^70^

An alternative approach for mining for phage peptidoglycan hydrolases (PGHs)–derived antimicrobial peptides was proposed by Wu et al.^71^ The study introduced a computational pipeline to mine AMPs derived from ESKAPE microbes (a group of clinically dangerous pathogens comprising *Enterococcus faecium, Staphylococcus aureus, Klebsiella pneumoniae, Acinetobacter baumannii, Pseudomonas aeruginosa* and *Enterobacter* spp) and their associated phages. To evaluate the antibacterial activity of the extracted peptides, the authors trained a model with CNNs and LSTM layers, basing on the model used by Ma et al.^5^ The result is a database, ESKtides, containing over 12 million peptides with predicted high antibacterial activity.

### AMP Mining of the Microbiome

Using the human gut microbiome as the biological sequence resource, Ma et al.^5^ mined for AMPs using deep learning techniques, including LSTM, attention, and BERT. The study identified 181 peptides showing antimicrobial activity, many of which had less than 40% sequence homology to known AMPs, demonstrated significant efficacy against antibiotic-resistant, Gram-negative bacteria, and in reducing bacterial load in a mouse model of lung infection.

In another study, deep learning discriminator methods were applied for the task of anticancer peptide (ACP) prediction, leveraging the overlap between ACPs and AMPs.^72^ The study employed a high-throughput mining process to identify 40 potential ACPs from the gut microbiome metagenomic data. Out of these, 39 peptides showed significant anticancer activity in various cancer cell lines. Two peptides, in particular, demonstrated exceptional efficacy in reducing tumor size in a mouse model without causing toxicity.

Furthermore, nearly a million new potential AMPs were recently discovered by computational analysis of the global microbiome.^3^ Using machine learning, the authors explored the vast diversity of the microbial world, analyzing 63,410 metagenomes and 87,920 microbial genomes. Additionally, identification of the peptides in proteomics and transcriptomics data was used as a filtering step after identification based on genomic sequence, The study resulted in the computational prediction of nearly a million candidates for new AMP, which were deposited in the AMPSphere database.

Another study integrated metagenomes from four different body sites to identify smORF-encoded peptides.^58^ To evaluate smORFs that were likely to encode AMPs, the authors used a random-forest based discriminator model. The study identified 323 candidate antibiotic peptides, showing activity against clinically relevant pathogens, both *in vitro* and *in vivo*.

Finally, several recent studies turned to mining microbiomes other than human gut, focusing on particularly promising species that are known to effectively maintain microbiome homeostasis.^73,74^ For example, Klimovich et al. performed high-throughput transcriptome and genome sequencing, followed by machine learning-based analysis of freshwater polyp *Hydra*’s microbiome. The study revealed that AMP-encoding genes underwent a recent, rapid evolution in the Hydra species, that AMPs are selectively expressed in certain cell types, and finally, that the AMP activity follows a spatial pattern, suggesting that depending on the microhabitat different cocktails of AMPs targeting different bacterial species are secreted to generate a specific chemical landscape to locally control the density and shape the composition of the microbiome of *Hydra*.^73^ Another study, based on a deep learning model with Dense-Net blocks and a self-attention module,^74^ focused on the gut microbiome of cockroaches, which harbors harmful species without occurring pathogenesis.

### Exhaustive Mining of Combinatorial AMP Sequence Spaces for Short Peptides

Instead of mining natural biological sequence resources, recent efforts focus on evaluating all possible sequences of up to a fixed, short length. Huang et al. developed a machine-learning-based pipeline to systematically identify effective AMPs from a vast virtual library of peptides made of 6–9 amino acids.^75^ Their pipeline consists of multiple sequential machine-learning modules designed to filter, classify, rank, and predict the efficacy of potential AMPs. Since their discriminator models were trained on the GRAMPA data set,^76^ a compiled collection of MIC measurements that could suffer from lab-specific biases, the authors adopted a two-step experimental validation strategy, refining their discriminator after the initial stage to mitigate possible biases in the training data. The results of the study include the identification of three potent hexapeptides that exhibit strong antimicrobial activities against multidrug-resistant pathogens, comparable efficacy to penicillin in treating bacterial infections in mice, and low toxicity. Another study focused on developing AMPs against *Acinetobacter baumannii*, scanning through the entire libraries of hexapeptides, heptapeptides, and octapeptides, encompassing tens of billions of candidates. Their pipeline included classifiers for *A. baumannii*-specific AMPs, trained on an extremely scarce training data set of only 148 sequences using a few-shot learning strategy involving pretraining and multiple fine-tuning steps.

## AMP Generation

Generative AI holds the promise to transform the discovery of novel drug candidates.^79^ By learning and modeling underlying data distributions, generative AI may become indispensable tools for peptide generation in the future. Several generative AI methods have already been applied for this purpose, showing promising results and paving the way for future advancements in peptide-based antimicrobial drug discovery.^1,15,80,81^

### Modeling Frameworks Employed in AMP Generation

The generative AI approaches applied so far differ by the specific modeling framework that they use (Table 3). While the use of autoregressive models, such as LSTMs and more generally RNNs,^29,82^ has been explored, they are currently less frequently used compared to other methods. The majority of research thus far has focused on the implementation of variational autoencoders (VAEs)^1,25,83–88^ or Wasserstein autoencoders.^80,89^ Generative adversarial networks (GANs)^90–94^ have also seen significant application, demonstrating their ability to generate new AMP sequences. Most AMP generation methods focus on generating candidates with promising antimicrobial candidates, with a smaller number addressing hemolytic or cytotoxic properties as well. While some generative AI studies included microbiological testing of generated AMP candidates, relatively few were further tested in animal models (Table 3). The different model architectures and their training processes have been discussed in detail in our recent review.^95^

**Table 3:** Generation Approaches to AMP Discovery

method	generation mode	controlled generation	aimed properties	generation framework	experimental validation	MD
AMP-GAN^93^	unconstrained	conditional generation	sequence length, microbial target, target mechanism, activity	cGAN	microbiological assays, cytotoxicity assays	yes
MMCD^102^	unconstrained	conditional generation, contrastive learning	AMP, ACP	diffusion		
CLaSS^80^	unconstrained	discriminator-guided filtering	AMP, activity, nontoxicity, structure	WAE	microbiological assays, *in vivo* animal models, cytotoxicity assays, hemolysis assays	yes
LSSAMP^83^	unconstrained	latent space sampling	secondary structure	vector quantized VAE	microbiological assays, *in vivo* animal models, cytotoxicity assays, hemolysis assays	
AMP-Diffusion^101^	unconstrained	positive-only learning	AMP	PLM + diffusion	microbiological assays, *in vivo* animal models, cytotoxicity assays	
AMPGAN v2 ^94^	unconstrained	conditional generation	sequence length, microbial target, target mechanism, activity	cGAN		
AMPTrans-LSTM^82^	unconstrained	discriminator-guided filtering	AMP	LSTM + transformer		
Zeng et al.^99^	unconstrained	discriminator-guided filtering	AMP	PLM	microbiological assays	
Jain et al.^96^	unconstrained	active learning	AMP	GFlowNets + active learning		
Pandi et al.^24^	unconstrained	discriminator-guided filtering	activity	VAE	microbiological assays, cytotoxicity assays, hemolysis assays	yes
M3-CAD^85^	unconstrained	conditional generation, discriminator-guided filtering	microbial target, nontoxicity, mode of action	cVAE	microbiological assays and *in vivo*, cytotoxicity assays, hemolysis assays	
Ghorbani et al.^88^	unconstrained		AMP	VAE		
MODAN^97^	optimized	bayesian optimization	Activity and nontoxicity	Gaussian process	microbiological assays, hemolysis assays	
Cao et al.^92^	unconstrained	discriminator-guided filtering	AMP	GAN	microbiological assays	yes
Diff-AMP^100^	unconstrained	discriminator-guided filtering	AMP	Diffusion		
HydrAMP^1^	unconstrained, analogue	conditional generation	AMP, activity	cVAE	microbiological assays, hemolysis assays	yes
AMPEMO^98^	optimized	discriminator-guided filtering	AMP, diversity	Genetic algorithm		
Buehler et al.^103^	unconstrained	conditional generation	secondary structure, solubility	GNN		
Renaud and Mansbach^84^	unconstrained, analogue	latent space sampling	AMP, hydrophobicity	VAE		
Capecchi et al.^29^	unconstrained	discriminator-guided filtering, positive-only learning	activity, nontoxicity	RNN	microbiological assays, hemolysis assays	
Multi-CGAN^90^	unconstrained	conditional generation	activity, nontoxicity, structure	cGAN		
QMO^89^	optimized	zeroth-order optimization, gradient descent	activity, nontoxicity	WAE		
PandoraGAN^91^	unconstrained	positive-only learning	antiviral activity	GAN		
PepVAE^86^	unconstrained	latent space sampling	activity	VAE	microbiological assays	
ProT-Diff^104^	unconstrained	discriminator-guided filtering, positive-only learning	AMP, activity	PLM + diffusion	microbiological assays and *in vivo*, cytotoxicity assays, hemolysis assays	
MOQA^87^	optimized	D-wave quantum annealer	activity, nontoxicity	binary VAE	microbiological assays, hemolysis assays	

### Controlled AMP Generation

Generative AI methods can efficiently produce thousands of plausible peptide candidates, making it crucial to direct the generation process toward acquiring desired properties to increase the likelihood of identifying relevant hits. One fundamental approach to controlled generation is the use of auxiliary discriminators to guide the generative process and to filter top candidates. In the CLaSS model,^80^ discriminative models are trained on the latent space of a WAE, guiding the generation toward peptides with targeted activity and toxicity. Another straightforward strategy is based on positive-only learning, as demonstrated by PandoraGAN,^91^ where only highly active peptides are used for training. However, both discriminator-guided filtering and positive-only learning are limited by the sparse availability of positively labeled training data, namely active and nontoxic AMPs.

The advancement of GAN or VAE-based models has led to the development of their conditional variants, such as cGANs^90,93,94^ and cVAEs.^1,85^ These models are configured during the generation phase to produce peptides more likely to meet specific criteria. For instance, Multi-CGAN^90^ optimizes the generation process to address multiple properties simultaneously, while M3-CAD,^85^ a multimodal, multitask, and multilabel cVAE, targets eight feature categories including predicted 3D structure, species-specific antimicrobial activities, antimicrobial mechanisms, and toxicity.

Additionally, some methods exploit the model’s latent space to guide generation. Techniques such as latent space sampling allow for the selection of peptides from regions expected to encode desirable attributes.^83,84,86^ For example LSSAMP^83^ discretizes the latent representation to encode both sequence and structural information, facilitating the generation of peptides with desired secondary structures.

To tackle challenges like training data deficiency, the model of Szymczak et al., called HydrAMP, introduced several key enhancements to the standard cVAE framework.^1^ The model focused on generating highly active antimicrobial peptides (AMPs) by conditioning on properties like low MIC values. HydrAMP included a pretrained classifier to ensure that the generated peptides retained the desired properties. To improve training stability, the authors added terms to the loss function that made sure the generated peptides closely matched the input and that the latent representations of the input and the generated peptides matched as well. HydrAMP also featured the ability to modify an existing peptide to meet specific activity conditions, controlled by a creativity parameter. Higher creativity led to more diverse analogues. Unlike standard cVAEs, which generate peptides by sampling from the latent space, HydrAMP could improve both known AMPs and peptides experimentally proven to lack antimicrobial activity. Molecular dynamics (MD) simulations provided additional descriptors of peptide activity, which, combined with a classifier ensemble, helped rank candidates for experimental testing. Finally, the most promising peptides were synthesized, and their activity and toxicity were experimentally validated. Using HydrAMP, Szymczak et al. discovered 15 novel, highly potent AMPs, that were active against several strains of bacteria, including multi drug resistant strains.

Analog generation, as employed by HydrAMP, is one way of lead optimization for AMPs, as it produces peptides similar to a nonactive prototype but with enhanced properties. A promising research direction for idealistic peptide design is direct optimized generation, often using tailored cost functions. In this domain, QMO^89^ uses zeroth-order gradient optimization to navigate the latent space. Other approaches addressing the optimization challenge include active learning with GFlowNets,^96^ quantum annealing,^87^ Bayesian optimization,^97^ and evolutionary algorithms.^98^

### Large Language Models Applied in AMP Generation

With the current success of tools such as chatGPT, generative language modeling becomes popular also in the field of AMP generation. While pretrained LMs are widely used in discriminative tasks, their application to AMP generation has been limited. AMP generation from pLMs typically involves either decoder-like architectures such as GPT^99^ or a diffusion process trained on continuous embeddings obtained from pretrained LMs.^100,101^ Unfortunately, these methods have so far implemented relatively simplistic strategies for controlled design, relying mainly on either positive-only learning or discriminator-guided filtering.^99–101^ A promising direction has been the use of contrastive learning, as in MMCD^102^ where training of a diffusion-based model involves contrasting the embeddings of known positive AMP examples with negative ones.

## Challenges and Prospects for the Future

With the AI revolution transforming the world today, there is growing potential for AI to enhance the design of novel AMP-based antibiotics and to fight antimicrobial resistance. Indeed, many breakthroughs have been made in recent years.^1,2,4,5,15,19,20,74^

The two described approaches: AMP mining, and AMP generation, equipped and enhanced by the use of cutting edge discriminative methods, are among the most promising strategies for AI-driven AMP discovery. However, limitations of existing tools in this domain need to be addressed, and open avenues of research remain unexplored.

### Challenges to Be Addressed in the Realm of Discriminative Models

Despite intensive development of discriminative models, challenges remain regarding their application in the AMP field. First, the development of predictive methods is hindered by the relatively small volume of available data. Recent approaches based on transfer learning, with particularly wide usage of pretrained and fine-tuned LLMs, promise to partially solve the problem of data scarcity. More developments for computational handling of low-data regimes, as well as initiatives enhancing data sharing and experimental validation efforts are needed as the data for multiresistant strains will remain insufficient for training strain-specific activity predictors. Finally, more emphasis should be placed on developing methods predicting peptides’ toxicity, a major barrier to the clinical application of AMPs. Currently, due to limited training data, existing methods for hemolytic activity prediction perform suboptimally, while cytotoxicity prediction methods are severely lacking.

Another major challenge is the lack of experimentally validated negative examples, which is easily explained by the lack of incentive to generate negative data.^105^ Moreover, a peptide may falsely appear negative if not tested against a sensitive strain or due to technical issues such as clumping in solution which can easily be mistaken for lack of activity. The lack of well-defined negatives poses a particularly difficult problem in the supervised learning paradigm. As shown by Sidorczuk et al.^106^ negative data set construction heavily influences the performance of models in the task of AMP prediction. Some approaches try to address the issue by modifying the loss function, for example by using asymmetric loss that down-weights the negative samples,^53^ or by adapting the data sampling procedure, for example down-sampling the negative examples.^30,48^ Additionally, different studies determine both positives and negatives under different experimental conditions, such as medium and bacterial concentrations, further adding confusion to the definition of labels for model training.^15^ Therefore, databases of AMPs should be populated by researchers not only with the experimentally validated positives, but also negatives, and standards for the experimental conditions should be unified.

In addition, full use of peptide structural information could be more effective for functional prediction. However, due to scarce structural information, in-depth analysis of the importance of structure formation for AMP property prediction is currently limited. Moreover, the structures available in the databases such as DBAASP are obtained without taking into consideration neither the proximity of the cell membrane nor the effect of self-association of the peptides into larger aggregates. Additional structural information such as secondary and tertiary structures and post-translational modifications could be considered in future studies, providing an opportunity to improve the performance of AMP predictions.

Moreover, it is crucial to verify the robustness and generalizability of the model on different independent data sets. However, existing deep learning models have not yet been objectively evaluated using external data sets, which should be addressed.^35^

While quite successful at classification of linear peptide data, current discriminative methods are not applicable to a wide realm of modified peptides. A significant subset of antimicrobial peptides contains noncanonical building blocks such as cycles, β-amino acids, modified cysteines, and lipid attachments, distinguishing them from purely linear peptides, however those types of peptides are not abundant enough for AI classifier training^107^ Existing FDA-accepted AMPs, such as polymyxins, significantly differ from such linear peptides.^108^ This limits the applicability of discriminative methods for detection of clinically relevant AMPs. Therefore, more data on complex and modified peptides should be deposited in dedicated databases to enable AI model training.

Furthermore, data on other important properties of peptides could be used in the future to train better classifiers. For example, peptides can be degraded *in vivo*, resulting in half-lives that are insufficient for translational development. Currently, relevant training data on half-lives is not available and would greatly benefit AI-driven design. Such data could be collected by conducting systematic experimental studies that measure peptide stability in various biological environments, and compiling the results into publicly accessible databases for machine learning applications. To our knowledge, there is also no available data about ADMET properties of AMPs, which could be used to train AI models. To address this challenge, a large experimental study testing ADMET properties of peptides would be required. Alternatively, as performed by Mishra and Muthukaliannan et al.,^109^ machine learning approaches for predicting ADMET properties of small molecules could be applied to SMILES representations of peptides. However, such predictions may be unreliable due to the larger size and different physicochemical properties of peptides compared to small molecules.

### Challenges to Be Addressed in AMP Mining

AMP mining in biological sequences brings the advantages of identifying highly realistic peptides as AMP candidates. Indeed, the biological peptide sequences contain only L-amino acids and thus can be easily synthesized by solid phase synthesis with low costs and limited byproduct reactions. AMPs secreted in living organisms, such as in the gut microbiome, may specifically target invading microbes while avoiding toxicities to hosts. However, the potential of biological sequence mining strategies for discovering AMP candidates is realized only under specific conditions: first, AMPs with high activity and low toxicity must be present within the biological sequences being mined; second, the discriminative methods used must be capable of accurately identifying these AMPs. While the existing mining approaches have already proven successful,^2,3,5,19,74^ these conditions underline the necessity of having both a rich biological data set and robust discriminative models. Furthermore, the genomic context has long been recognized as a critical factor in functional predictions of biological sequence properties.^110^ Recent advancements have seen techniques from natural language processing being innovatively applied to gene function prediction, providing new ways to interpret complex biological data.^111–113^ Adapting these approaches in the context of AMP mining could allow for better use of the limited labeled data for AMPs, but testing their accuracy empirically remains an open problem.

AMP mining could also benefit from expanding the analytical framework beyond genomics and proteomics alone by integrating additional data types into the predictive models. These could include transcriptomics data, which has been shown to be predictive of protein function^114^ or ribosomal sequencing data.^115^ However, there is still a shortage of data sets with these data types compared to the widespread availability of genomes.

While some peptides are produced by a complex process of post-translational modifications or even nonribosomally, many peptides are encoded by single genes, which can be directly transcribed and translated, or derived from the cleavage of a single precursor protein. This simpler genetic architecture makes peptides particularly amenable to exploitation for biotechnological purposes. Exploiting the potential of biosynthetic gene clusters, which must be expressed as a group to produce the desired compound^116,117^ could prove beneficial for AMP discovery.

Moreover, processing each sequence independently with a pretrained model, although practical, overlooks valuable information contained in natural variations. Where multiple sequences are available,^118^ employing models that analyze multisequence alignments rather than individual sequences can offer significant benefits.

Finally, since AMP mining relies on discriminative methods, their limitations transfer naturally to this approach. In particular, as discriminative methods cannot detect complex, chemically modified peptides, currently only linear peptides can be mined. Although recent AMP mining studies did perform preclinical testing of the most promising AMP candidates in animal models, none of those candidates went further to clinical studies yet.

### Challenges to Be Addressed in AMP Generation

Similarly to AMP mining, generative AI has the potential to accelerate AMP discovery, but several obstacles remain. First, evaluation and benchmarking of generative models prove difficult. Generated peptides are most often evaluated with respect to diversity, novelty, and similarity to training data, but the activity and toxicity remain unknown except for a small experimentally validated subset. Auxiliary discriminators are used to estimate those properties of interest, but the choice of such models is entirely arbitrary, making comparison between models impossible. Second, as generative methods are capable of generating thousands of candidates within a short span of time, efficient methods to rank top candidates are needed. Currently, top candidates are selected using extensive filtering and expert knowledge. The existing generative approaches are also limited in their performance due to low data availability. Additionally, searching for potent peptides can be thought of as generating examples out-of-distribution, a widely recognized problem in generative modeling.

With few exceptions,^97^ generative AI methods operate only on the 20-letter amino acid alphabet, without taking into account post-translational modifications or nonstandard amino acids. Thus, they are unable to sample from the huge space of peptides with chemical modifications, thereby largely underestimating the full complexity of the peptide world. Further extensions of the generative models to account for nonstandard amino acids may result in highly potent AMP designs in the future. So far, there is not enough training data to equip generative AI with the abilities to directly design such complex peptides as those currently in use in the clinic. However, the promising linear peptide candidates can be further enhanced by rational design, increasing their stability, efficacy and safety by choosing from the repertoire of typical chemical modifications, such as cyclization, residue phosphorylation or addition of lipids.^12^

In comparison to AMP mining, relatively fewer AMPs obtained from generative AI methods were confirmed preclinically *in vivo*, which may stem from the fact that the majority of AI laboratories have limited experimental capacities. Therefore, AI-discovered AMPs are yet to be tested in clinical trials. This calls for collaborative efforts of AI, chemical and biological laboratories joining forces with industrial partners to cover the steps from discovery to the clinics.

Finally, the emerging generative AI methods are usually benchmarked and developed for generation of text or images and are not always well-suited for the generation of peptides. Importantly, most existing generative AI models need specific modeling extensions to achieve controlled generation, which poses an important research direction that is largely unexplored for the design of AMPs.

## Summary

The future of antimicrobial peptide discovery is on the cusp of a transformative revolution with the integration of AI technologies. Since early pioneering work demonstrated that machines could design peptide antibiotics effective in preclinical mouse models,^20^ this field has grown and matured significantly. AI-driven approaches have already dramatically accelerated our ability to identify new AMPs. By leveraging large-scale genomic and proteomic data, coupled with sophisticated generative and discriminative models, AI will facilitate the design of potent AMPs specifically tailored to combat emerging resistant pathogens. This synergy between AI and biotechnology promises to accelerate the drug discovery process while also overcoming limitations associated with traditional approaches.
